# Host inflammatory response to polypropylene implants: insights from a quantitative immunohistochemical and birefringence analysis in a rat subcutaneous model

**DOI:** 10.1590/S1677-5538.IBJU.2015.0289

**Published:** 2016

**Authors:** Alessandro Prudente, Wágner José Fávaro, Paulo Latuf, Cássio Luis Zanettini Riccetto

**Affiliations:** 1 Faculdade de Ciências Médicas da Universidade de Campinas, Campinas, SP, Brasil;; 2 Instituto de Biologia, Universidade de Campinas, Campinas, SP, Brasil;; 3 Laboratório de Investigação Patologica, Centro de Investigação em Pediatria,Universidade de Campinas, Campinas, SP, Brasil

**Keywords:** urinary incontinence, pelvic organ prolapse, polypropylenes, graft versus host reaction, foreign body reaction, immunohistochemistry

## Abstract

**Objectives:**

To describe acute and sub acute aspects of histological and immunohistochemical response to PP implant in a rat subcutaneous model based on objective methods.

**Materials and Methods:**

Thirty rats had a PP mesh subcutaneously implanted and the same dissection on the other side of abdomen but without mesh (sham). The animals were euthanized after 4 and 30 days. Six slides were prepared using the tissue removed: one stained with hematoxylin-eosin (inflammation assessment); one unstained (birefringence evaluation) and four slides for immunohistochemical processing: IL-1 and TNF-α (pro-inflammatory cytokines), MMP-2 (collagen metabolism) and CD-31 (angiogenesis). The area of inflammation, the birefringence index, the area of immunoreactivity and the number of vessels were objectively measured.

**Results:**

A larger area of inflammatory reaction was observed in PP compared to sham on the 4th and on the 30^th^ day (p=0.0002). After 4 days, PP presented higher TNF (p=0.0001) immunoreactivity than sham and no differences were observed in MMP-2 (p=0.06) and IL-1 (p=0.08). After 30 days, a reduction of IL-1 (p=0.010) and TNF (p=0.016) for PP and of IL-1 (p=0.010) for sham were observed. Moreover, area of MMP-2 immunoreactivity decreased over time for PP group (p=0.018). Birefringence index and vessel counting showed no differences between PP and sham (p=0.27 and p=0.58, respectively).

**Conclusions:**

The implantation of monofilament and macroporous polypropylene in the subcutaneous of rats resulted in increased inflammatory activity and higher TNF production in the early post implant phase. After 30 days, PP has similar cytokines immunoreactivity, vessel density and extracellular matrix organization.

## INTRODUCTION

Since the introduction of synthetic mesh implants for tissue reinforcement, surgical treatment of urinary incontinence pelvic floor prolapse has changed. Success rates have increased and became long lasting, however, harmful events related to biomaterials integration have been observed, even for organic or synthetic meshes (1-4). Adverse reactions related to synthetic mesh implants include chronic pain, dyspareunia, urinary or vaginal erosion of the mesh as well as lower urinary tract symptoms (5, 6).

Polypropylene (PP) is currently the most common material used in pelvic floor reconstructive surgery and stress urinary incontinence treatment. It is a hydrophobic and non-hydrolyzable polymer derived from oil refining. Sterilization is undertaken by either heat or radiation thus promoting molecular structural changes of the original polymer. Therefore, the biological response is not only a consequence of the contact between host and polymer, but is also a result of chemical changes in the preparation process (7). Several mechanical and histological characteristics secondary to PP implants in living organisms have been demonstrated (8-10). Most of the histological and immunohistochemical evaluations are based on the description of cellular types and/or semi-quantitative measurements of their distribution in randomized samples (11, 12). There is no standard way to study these implants. It should include objective, reliable and reproducible techniques that consider histological, cellular, molecular and even genetic aspects of this host response.

The aim of the present study was to describe acute and sub-acute aspects of histological and immunohistochemical response to PP implant in a rat subcutaneous model based on objective quantification methods.

## MATERIALS AND METHODS

The study followed the ethical principles for animal experiments adopted by the Brazilian College of Animal Experiments and was carried out after approval by the Ethics Committee for Animal Experiments of the Institute of Biology of the University of Campinas, Brazil (protocol 2400-1).

The mesh used in this study is made of monofilament type I polypropylene with an original weight: 44g/m^2^ and pores of 1mm and was the same as that included in NAZCA TC^TM^ and Calistar A^TM^ sets (Promedon™-Cordoba, Argentina), currently commercially available. Meshes were provided by the company in single sterilized packs and were sterilized using ethylene oxide.

### Surgical procedure and tissue preparation

Thirty female, eight week old Wistar rats, weighing between 150 and 200g, received on one side of their abdominal wall an implant of a 10x10mm monofilament PP mesh.

After anesthesia with sodium pentobarbital 3% (0.15mg/g), a 2-cm cross-sectional incision was made in the lower abdominal region. The mesh was implanted in the animal in a standardized manner on one side of the abdominal wall between the hypodermis and the anterior fascia of the abdominal musculature. A similar dissection was then carried out on the other abdominal side but without mesh implant (sham). The animals were divided into two groups of 15 animals which were euthanized on the 4^th^ and the 30^th^ day after mesh implantation with a lethal dose of sodium pentobarbital 3%.

The whole abdominal wall was immediately removed for analysis and the sham areas and those with the implants were fixed (formalin 10% for 24 hours). Three consecutive sections of 5μm thickness were then placed on each of six slides; one stained slide with hematoxylin-eosin for optical microscopy (inflammation assessment); one unstained slide for polarization microscopy analysis (collagen fibers birefringence evaluation) and four slides for immunohistochemical processing with the following antibodies: anti-CD-31 (angiogenesis), anti-interleukin 1(anti-IL-1) and anti-tumor necrosis factor (anti-TNF-α) (inflammation and cytotoxicity) and anti-metalloproteinasis-2 (anti-MMP-2) (collagen metabolism).

### Histologic evaluation

**Figure 1:**
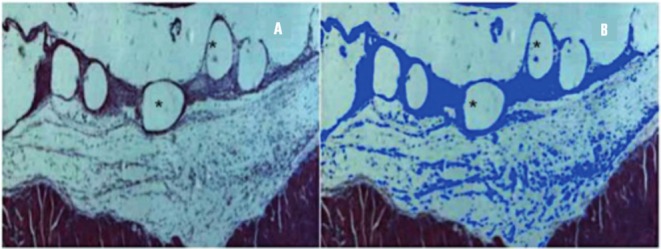
Evaluation of the inflammatory reaction. (A) Inflammatory tissue around the PP filaments (rounded blank areas-*)-HE/100x; (B) Blue marks represent inflammatory reaction after processing by Axiovision software™-HE/100x

Inflammatory reaction (hematoxylin-eosin staining) was studied on the 4^th^ and 30^th^ days post implant. The same researcher analyzed all slides, although he had not had knowledge of what animal or fragment was evaluating. On each slide, three photomicrograms (200x magnification) of the implant site were recorded. Axio Vision™ V 4.8.0.0 software (Karl Zeiss, Jena, Germany) was used to select and measure the areas of inflammatory reaction around the polypropylene filaments, as showed in Figure-1.

### Birefringence analysis

**Figure 2:**
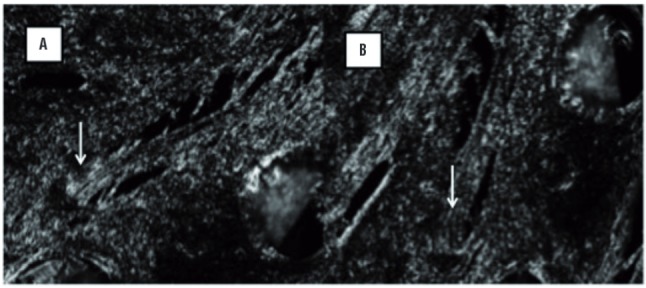
Evaluation of collagen fibers birefringence in polarization microscopy. (A) The collagen fibers bright on the dark background. (B) The same area after 45o rotation of the polarization microscope stage. White arrow indicates the same fiber package in opposite arrangement (200x).

The analysis of the direction and packing of collagen fibers was performed by polarizing microscopy, but only for those animals euthanized at 30 days due to the time necessary for the growth of the host collagen fibers. In each selected image (200x magnification), two records were obtained by rotating the microscope stage at approximately 45º in each field, to obtain the maximum polarization effect (called positions A and B), in order to demonstrate the group of fibers with the same or opposite orientation (Figure-2).

A ratio was calculated using the percent area of fibers from the same field, identified as birefringence index. This was obtained by dividing the percent birefringent area measured in the position A by the percent birefringent area measured in the position B (index A/B). Low ratios, close to 1 (one), indicate fibers with a similar birefringence in the two positions, reflecting a disorderly orientation. Therefore, the higher the index A/B, the higher the organization of collagen fibers in the same direction. The intensity of brightness (pixel/μm^2^) emitted by collagen fibers was also evaluated, in order to estimate collagen density and packing.

### Immunohistochemical analysis

Tissue specimens fixed in 10% formalin and embedded in paraffin were sectioned and placed on silanized slides. After initial processing, sections were incubated at room temperature for 30 min and then overnight at 8ºC with mouse monoclonal antibodies to CD31 (clone JC/70A, ab 9498, Abcam™, diluted 1/100) and polyclonal antibodies to IL-1 (ab106035, Abcam™,diluted 1/1000), TNF Receptor I (ab19139, Abcam™,diluted 1/1000) and MMP2 (ab37150, Abcam™,diluted 1/250). All antibodies were diluted with Dako Antibody Diluent (S3022, Dako™). Antigen-antibody binding was detected using the Advance system (K4068, AdvanceHPR, Dako™), and immunostaining was achieved using diaminobenzidine (K3468, Liquid DAB+substrate Dako™). Internal positive controls, as well as positive cases were previously used. Negative controls were represented by the same tissue sample used for positive control, in which the primary antibody was omitted.

The immunohistochemical analysis was carried out using specific antibodies to evaluate: (a) pro-inflammatory cytokines (interleukin-1–IL-1 and tumour necrosis factor-alpha-TNF-α); (b) collagen metabolism (metalloproteinase 2-MMP-2) and (c) angiogenesis (surface antigen CD-31).

**Figure 3:**
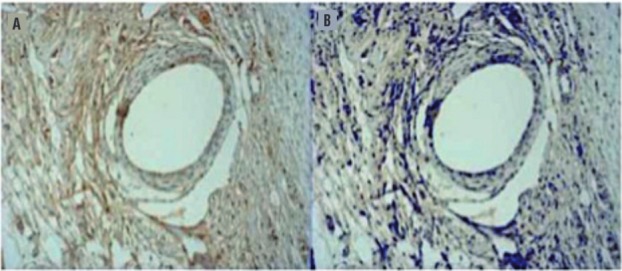
Example of MMP2 immunoreactivity in an implant sample. Blank rounded area indicates the PP filament. (A) Before software selection. (B) After selection, note MMP2 immunoreactivity colored in blue (200x).

A Primo Star^TM^ Zeiss microscope (Carl Zeiss Microscopy, Jena, Germany) was used for histological evaluation. The entire slide was scanned using a 200x magnification (400x for vessel density), and three fields for each slide were randomly selected for subsequent image acquisition using a Zeiss AxioCam camera ICC1^TM^. Objective analysis of immunoreaction (percentage area of immunoreactivity and vessel density) was carried out with AxioVision V 4.8.0.0 Software Microscope (Karl Zeiss-Germany) (Figure-3).

### Statistical Tests

The Kruskal-Wallis test was performed for comparisons between periods and the Wilcoxon test for comparisons between groups. For repeated measures the ANOVA was used for comparisons of groups and periods. A 5% significance level was adopted for all statistical tests (p<0.05).

## RESULTS

All rats survived and no complications were observed during the post implant period. In addition, no dehiscence or mesh exposure at the implant site was observed.

### Histological analysis

Histological analysis of implantation site showed an expected pattern of acute inflammatory reaction at four days based on macrophages and polymorphonuclear infiltrate with few fibroblasts and edema. However, on the 30^th^ day post implant, a foreign body reaction based on histiocytes and giant cells was the predominant pattern around the PP filaments, combined with many fibroblasts with intense production of collagen resulting in a compact tissue. A larger area of inflammatory reaction was observed in the PP group compared to sham on the 4^th^ and also on the 30^th^ day (11.36% (PP) x 5.19% (sham) and 11.06% (PP) x 5.73% (sham) for 4 and 30 days respectively, p=0.0002) but no differences were observed when comparing the different times (4 and 30 days) in each one (sham and PP groups) (Table-1).

**Table 1:** Inflammatory reaction (mean percent area).

	PP (SD)	Sham (SD)
4 days*	11.36 (6.94)	5.19 (1.68)
30 days*	11.06 (6.85)	5.73 (1.97)

*p = 0.0002 (sham x pp) **SD** = Standard deviation

### Birefringence analysis

The analysis of the organization of collagen fibers, represented by the birefringence index (index A/B), showed no differences between PP and sham (1.29 (PP) x 1.43 (sham), p=0.27). The collagen density (which estimates the intensity of tissue compaction) also showed no differences between the groups after 30 days post implantation (Table-2) (Figure-2).

**Table 2:** Birefringence analysis of collagen fibers.

	Position A			Position B			Index A/B		

	PP (SD)	Sham (SD)	p	PP (SD)	Sham (SD)	p	PP (SD)	Sham (SD)	p
Collagen fibers area (mean percent area)	8.37 (5.32)	10.65 (4.63)	0.24	7.78 (5.18)	11.60 (7.67)	0.24	1.29 (0.24)	1.43 (0.33)	0.27
Collagen density (mean pixel/μm^2^)	73.58 (48.32)	48.37 (4.42)	1	79.15 (56.35)	50.44 (5.17)	0.73	-	-	

**SD = Standard deviation**

### IL-1

**Figure 4:**
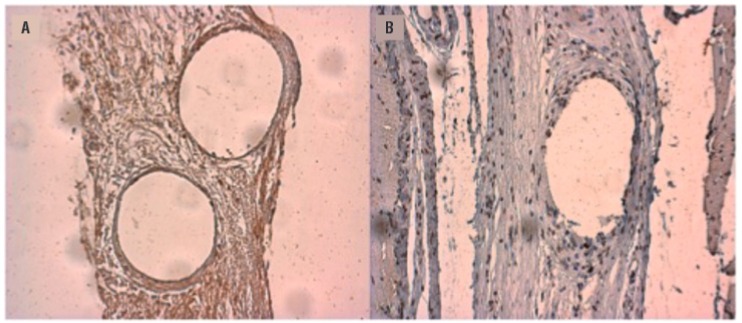
Example of Il-1 immunoreactivity (Brown area) after 4 days (A) and 30 days (B)–(200x). Note a higher brown intensity and extension in A.

A reduction of IL-1 immunoreactivity was observed after 30 days post implant when compared with 4 days for both groups (50.07% (4 days) x 25.66% (30 days) and 32.36% (4 days) x 27.09% (30days), for PP and sham respectively, p=0.010) (Figure-4). On the 4^th^ day, PP presented a slightly higher but not significant level of IL-1 immunoreactivity than the sham (p=0.08).

### TNF-α

A higher TNF-α immunoreactivity in PP group was observed when compared with sham on the 4^th^ day (56.42% (PP) x 31.98% (sham), p<0.0001). Comparing the features on the 4^th^ and the 30^th^ day for each group, there was a similar TNF-α immunoreactivity over time in sham group and it was observed a reduction over time in PP group (56.42% (4 days) x 40.65% (30days), p=0.0161) (Table-3).

**Table 3:** Immunohistochemistry analysis of angiogenesis, inflammation and collagen metabolism.

	IL-1*			TNF*			MMP-2*			CD-31**	

	PP (SD)	SHAM (SD)	p	PP (SD)	SHAM (SD)	P	PP (SD)	SHAM (SD)	p	PP (SD)	SHAM (SD)
4 days	50.07 (13.47)	32.36 (20.31)	0.08	56.42 (7.61)	31.98 (11.92)	<0.0001	54.19 (25.24)	31.65 (9.07)	0.066	N/A	N/A
30 days	25.66 (14.41)	27.09 (18.84)	0.08	40.65 (15.49)	34.39 (11.92)	0.420	29.98 (14.77)	44.57 (14.53)	0.024	19.64 (9.43)	16.54 (7.94)
p	0.010	0.010		0.0161	0.523		0.018	0.058		PP x SHAM	0.587

*Mean percentage of the area marked by the antibody relative to the field** Average number of vessels per field **SD** = Standard deviation

### MMP-2

PP group presented a higher MMP-2 immunoreactivity after 4 days compared to 30 days while sham presented similar levels over time (55.19% (4 days) x 29.98% (30 days), p=0.018) (Table-3). Sham presented higher MMP-2 immunoreactivity on the 30^th^ day (31.65% (4 days) x 44.57% (30 days), p=0.024) however no difference was observed in comparison to PP group on the 4^th^ day (p=0.066).

### CD-31

There were no differences in the average number of vessels per field between PP and sham at 30 days (19.64 (PP) x 16.54 (sham), p=0.587) (Table-3).

## DISCUSSION

In addition to this study, several others, albeit using different methods, have described histological and molecular changes after the implantation of biomaterials, in particular, polypropylene (8, 9, 10, 11). An inflammatory response to macroporous monofilament PP was demonstrated in explanted meshes from humans one year after implantation and it was observed that this material had little long-term influence on the extracellular matrix composition, represented by the fraction of collagen and elastin in the tissue. However, consistent high concentrations of mast cells and macrophages were observed, which may suggest the perpetuation of a mild inflammatory foreign body reaction (13). Vandervord et al. implanted four types of biological meshes in subcutaneous of mice and found that, after a period of 12 weeks, the swine intestinal submucosa (SIS) presented a more effective integration represented by a significantly thicker inflammatory capsule with increased angiogenesis. The authors concluded that the control of inflammatory reaction and angiogenesis are the basis for effective tissue integration of the implant (14). In this present study, inflammatory reaction, elicited by PP implant, although higher than sham, did not differ significantly over time. Moreover, both groups presented a similar number of vessels after 30 days.

The impact of changes on the weight of the PP mesh and its combination with polyglactin in the inflammatory reaction were tested in a macrophage culture. In spite of demonstrating higher apoptosis levels than those of sham, no differences in the apoptosis index were found between the meshes. Furthermore, a higher rate of cell proliferation in mesh samples was observed than in those of sham (15). The present study, using histological samples, also found a higher cell proliferation rate (higher inflammatory reaction area) in PP group than sham. These findings, as well as others in vivo (16) and in vitro (17), suggest that mesh composition in addition to its surface features might be as important (or even more important) than its weight as a foreign body reaction drive.

Comparing PP and xenogeneic dermal collagen meshes, Zheng et al. found that the production of anti-inflammatory cytokines after the PP implant, such as interleukin-10 (IL-10) and tumor growth factor (TGF), was lower than the collagen group. An increased release of pro-inflammatory cytokines was also identified, such as interferon (IFN) and tumor necrosis factor (TNF-α) in PP meshes after the first week of implant, followed by a marked reduction over time and reaching the basal levels after thirty days (18). Moreover, when compared with sham (surgery without mesh), PP expressed a higher TNF-α level 24 hours after implantation (19). In the present study, a similar behaviour was observed in respect to pro-inflammatory cytokines (IL-1 and TNF-α) in the PP group. This may explain an increased inflammatory reaction area observed in the PP group. In another study, human blood samples showed a significant, although heterogeneous, increase of TNF levels after contact with PP meshes. Therefore, personal differences in TNF expression among patients may explain why women who undergo a surgical procedure under similar conditions can present different outcomes, such as a higher incidence and severity of mesh integration defects (20).

A reduced collagen deposition was observed 21 days after subcutaneous PP implantation in TNF-knockout rats when compared to control (22). This finding is consistent with the larger capsule thickness observed in the present study after PP implantation compared to sham, since the increased production of TNF may rise the proliferation and activation of fibroblasts and thus augment the collagen deposition (21, 22). Wu et al. analysed the MMP-2 gene activity and identified a higher gene expression in fibroblasts in contact with the mesh when compared with that found in the tissues far from the implant. According to these authors, at the beginning of the inflammatory process, remodelling of the extracellular matrix is essential for the migration and activation of inflammatory cells (23). Therefore, the production of matrix metalloproteinases (MMPs) by fibroblasts is an indicator of early inflammatory activity. During the implant integration, MMPs seem to be important in the tissue remodelling process as well as in the permanent mild foreign body reaction elicited by the presence of a no absorbable implant (23, 24). During the integration process, a progressive reduction in MMP-2 activity is generally expected in the same proportion as the body’s adaptation to the biomaterial. This was the case in the present study. A higher MMP-2 immunoreactivity was observed in the tissue around the mesh filaments after 4 days when compared to 30 days of implantation. Furthermore, the PP presented a lower MMP-2 immunoreactivity than sham at 30 days, which may indicate a trend of acceleration of the extracellular matrix remodelling.

The variety and concentration of cytokines, cells and collagen fibers at the surgical site during the healing process determines which type of scar tissue will emerge in the implant area. A previous study has shown that the amount and organization of extracellular tissue can establish relationship between the scar pattern around the implant and its biomechanical properties, as tensile strength and stiffness (8). In the present study, we proposed the use of the birefringence index for the assessment of the organization of collagen fibers. Birefringence is an important property of some synthetic and natural macromolecules. In the field of cell biology, birefringence is an important and reliable instrument for the analysis of collagen supra molecular properties (25). No differences in birefringence index between PP and sham were found. The collagen density analysis, which indicates tissue compression, also showed no difference between the groups 30 days after implantation. Therefore, in the present study, the PP elicited a similar quality and organization of extracellular matrix and did not stimulate an over production of collagen tissue in comparison to the regular healing process. In another sham-controlled study of subcutaneous implanted collagen coated versus uncoated PP meshes, the authors, although using semi quantitative methods, also observed a higher fibroplasia and no differences in angiogenesis or collagen fiber organization were observed between sham and PP (12).

Pierce et al. implanted meshes (PP and swine dermis) in the abdomen and vagina of rabbits and found that vaginal tissues demonstrated higher rates of inflammation; higher neovascularization but lower fibroblast proliferation than the abdomen independent of which mesh was being studied. Moreover, the same proportional difference between meshes was found in the vagina and the abdomen (26).

Despite advances in the understanding of the molecular process and histological response to mesh implant, their clinical translation requires further evidence. Rechberger et al. measured cytokines in the blood of patients undergoing sling surgery and found no differences between patients with or without mesh vaginal exposure during the follow-up. Only IFN, measured preoperatively, was higher among patients with exposed meshes. Therefore, the authors suggested that some blood tests could be used as complication predictors (27).

The upside of this study was to confirm previous data regarding the histological and molecular pattern of biological response to PP, based on objective and original methods for quantitative measurements (14). There are, however, downsides, since the methods did not allow the differentiation of cell types in each phase of the inflammatory process or the consideration of the long term aspects since the last measurement occurred at 30th day. Moreover, we did not completely avoid a systemic bias once we have used the same animal to both groups (sham and implant). There is also a lack of quantitative measurement of anti-inflammatory cytokines or an evaluation of mesh shrinkage or contraction and its relations with the inflammatory reaction that should be added in future studies.

## CONCLUSIONS

The implantation of monofilament and macroporous polypropylene in the subcutaneous of rats resulted in increased inflammatory activity and higher TNF production in the early post implant phase. After 30 days, PP has similar cytokines immunoreactivity, vessel density and extracellular matrix organization, in addition to lower MMP-2 expression than sham. The evaluation of inflammatory reaction after mesh implant should be based on objective standardized methods.

## References

[1] Patel BN, Lucioni A, Kobashi KC (2012). Anterior pelvic organ prolapse repair using synthetic mesh. Curr Urol Rep.

[2] Ostergard DR (2012). Evidence-based medicine for polypropylene mesh use compared with native tissue vaginal prolapse repair. Urology.

[3] Feiner B, Jelovsek JE, Maher C (2009). Efficacy and safety of transvaginal mesh kits in the treatment of prolapse of the vaginal apex: a systematic review. BJOG.

[4] Stanford EJ, Cassidenti A, Moen MD (2012). Traditional native tissue versus mesh-augmented pelvic organ prolapse repairs: providing an accurate interpretation of current literature. Int Urogynecol J.

[5] Mistrangelo E, Mancuso S, Nadalini C, Lijoi D, Costantini S (2007). Rising use of synthetic mesh in transvaginal pelvic reconstructive surgery: a review of the risk of vaginal erosion. J Minim Invasive Gynecol.

[6] Cornu JN, Peyrat L, Haab F (2013). Update in management of vaginal mesh erosion. Curr Urol Rep.

[7] Sternschuss G, Ostergard DR, Patel H (2012). Post-implantation alterations of polypropylene in the human. J Urol.

[8] Siniscalchi RT, Melo M, Palma PC, Dal Fabbro IM, Vidal Bde C, Riccetto CL (2013). Highly purified collagen coating enhances tissue adherence and integration properties of monofilament polypropylene meshes. Int Urogynecol J.

[9] Yildirim A, Basok EK, Gulpinar T, Gurbuz C, Zemheri E, Tokuc R (2005). Tissue reactions of 5 sling materials and tissue material detachment strength of 4 synthetic mesh materials in a rabbit model. J Urol.

[10] Pierce LM, Grunlan MA, Hou Y, Baumann SS, Kuehl TJ, Muir TW (2009). Biomechanical properties of synthetic and biologic graft materials following long-term implantation in the rabbit abdomen and vagina. Am J Obstet Gynecol.

[11] Huffaker RK, Muir TW, Rao A, Baumann SS, Kuehl TJ, Pierce LM (2008). Histologic response of porcine collagen-coated and uncoated polypropylene grafts in a rabbit vagina model. Am J Obstet Gynecol.

[12] Pierce LM, Asarias JR, Nguyen PT, Mings JR, Gehrich AP (2011). Inflammatory cytokine and matrix metalloproteinase expression induced by collagen-coated and uncoated polypropylene meshes in a rat model. Am J Obstet Gynecol.

[13] Elmer C, Blomgren B, Falconer C, Zhang A, Altman D (2009). Histological inflammatory response to transvaginal polypropylene mesh for pelvic reconstructive surgery. J Urol.

[14] VandeVord PJ, Broadrick KM, Krishnamurthy B, Singla AK (2010). A comparative study evaluating the in vivo incorporation of biological sling materials. Urology.

[15] Weyhe D, Belyaev O, Buettner G, Mros K, Mueller C, Meurer K (2008). In vitro comparison of three different mesh constructions. ANZ J Surg.

[16] Weyhe D, Schmitz I, Belyaev O, Grabs R, Müller KM, Uhl W (2006). Experimental comparison of monofile light and heavy polypropylene meshes: less weight does not mean less biological response. World J Surg.

[17] Prudente A, Riccetto CL, Simões MM, Pires BM, Oliveira MG (2013). Impregnation of implantable polypropylene mesh with S-nitrosoglutathione-loaded poly(vinyl alcohol). Colloids Surf B Biointerfaces.

[18] Zheng F, Xu L, Verbiest L, Verbeken E, Ridder D, Deprest J (2007). Cytokine production following experimental implantation of xenogenic dermal collagen and polypropylene grafts in mice. Neurourol Urodyn.

[19] Chatzimavroudis G, Koutelidakis I, Papaziogas B, Tsaganos T, Koutoukas P, Giamarellos-Bourboulis E (2008). The effect of the type of intraperitoneally implanted prosthetic mesh on the systemic inflammatory response. Hernia.

[20] Schachtrupp A, Klinge U, Junge K, Rosch R, Bhardwaj RS, Schumpelick V (2003). Individual inflammatory response of human blood monocytes to mesh biomaterials. Br J Surg.

[21] Junge K, Binnebösel M, Rosch R, Otto J, Kämmer D, Schumpelick V (2009). Impact of proinflammatory cytokine knockout on mesh integration. J Invest Surg.

[22] Grotenhuis N, Bayon Y, Lange JF, Van Osch GJ, Bastiaansen-Jenniskens YM (2013). A culture model to analyze the acute biomaterial-dependent reaction of human primary macrophages. Biochem Biophys Res Commun.

[23] Wu M-P (2010). Regulation of Extracellular Matrix Remodeling Associated With Pelvic Organ Prolapse. Journal of Experimental & Clinical Medicine.

[24] Souza-Pinto FJ, Moretti AI, Cury V, Marcondes W, Velasco IT, Souza HP (2013). Inducible nitric oxide synthase inhibition increases MMP-2 activity leading to imbalance between extracellular matrix deposition and degradation after polypropylene mesh implant. J Biomed Mater Res A.

[25] Vidal Bde C, Mello ML (2009). Structural organization of collagen fibers in chordae tendineae as assessed by optical anisotropic properties and Fast Fourier transform. J Struct Biol.

[26] Pierce LM, Rao A, Baumann SS, Glassberg JE, Kuehl TJ, Muir TW (2009y). Long-term histologic response to synthetic and biologic graft materials implanted in the vagina and abdomen of a rabbit model. Am J Obstet Gynecol.

[27] Rechberger T, Jankiewicz K, Adamiak A, Miotla P, Chrobak A, Jerzak M (2009). Do preoperative cytokine levels offer a prognostic factor for polypropylene mesh erosion after suburethral sling surgery for stress urinary incontinence?. Int Urogynecol J Pelvic Floor Dysfunct.

